# Two new species in *Iris* series *Chinenses* (Iridaceae) from south-central China

**DOI:** 10.3897/phytokeys.161.55483

**Published:** 2020-09-15

**Authors:** Carol A. Wilson

**Affiliations:** 1 University and Jepson Herbaria, University of California, Berkeley, 1001 Valley Life Sciences Building, Berkeley, California 94720-2465, USA University of California Berkeley United States of America

**Keywords:** Daba Mountains, Guizhou Province, karst terrain, phylogeny

## Abstract

*Iris dabashanensis* C.A.Wilson, **sp. nov.** and *I. probstii* C.A.Wilson, **sp. nov.** from China are described and illustrated. Both species occur on grassy slopes in mountainous regions of south-central China. The former is known from the Daba Mountains in rocky, calcareous soils associated with shrubs or mixed conifer and hardwood forests, while the latter is known from a region of karst terrain beside rice fields or under pine woods in Guizhou Province. Molecular data resolves both species in series *Chinenses* in a subclade that also includes *I. odaesanensis*, while morphologically they are similar to *I. henryi*. These newly described species are two of four members of series *Chinenses* that occur in south-central China.

## Introduction

*Iris* series *Chinenses* G.H.M. Lawr. comprises several Asian species that have short rhizomes, stolons, and small, open flowers where petals and sepals are horizontal. Dykes (1913) included four species in his informal group (the Chinese group), Lawrence (1953) circumscribed the series to include five species, Mathew (1989) recognized six species, and BIS (1997) recognized seven species in the series (Table 1). The two new species are from south-central China where *I. henryi* Baker and *I. proantha* Diels also occur, although none of their distributions overlap. *Iris minutoaurea* Makino, *I. odaesanensis* Y.N. Lee, and *I. rossii* Baker occur in northeastern China and adjacent regions in Korea and/or Japan and *I. koreana* Nakai is endemic to Korea. A species often included in the series, *I. speculatrix* Hance, is widespread in central and southern China. A phylogenetic study of the genus (Wilson 2011) that included five species from series *Chinenses* resolved the series as monophyletic, however *I. speculatrix* was not sampled. Guo and Wilson (2013) resolved *I. speculatrix* outside of series *Chinenses* in their study on crested species from several lineages in *Iris*. Tillie et al. (2001) included *I. speculatrix* as well as two series *Chinenses* species, *I. minutoaurea* and *I. rossii*, in their preliminary molecular phylogenetic study on *Iris*. Tillie et al. (2001) resolved *I. minutoaurea* and *I. rossii* as sister species and *I. speculatrix* outside of series *Chinenses*. Mavrodiev et al. (2014) suggested at least 23 genera should be recognized in *Iris* s.l., including a genus representing series *Chinenses* species. Crespo et al. (2015) formalized the splitting of *Iris* and circumscribed species from series *Chinenses* in the genus *Zhaoanthus* M.B. Crespo, Mart-Azorín & Mavrodiev. *Iris speculatrix* was not included in analyses of Mavrodiev et al. (2014) but based on several shared morphological characters was included in *Zhaoanthus* by Crespo et al. (2015).

**Table 1.** Taxonomic treatments of *Iris* series *Chinenses*. Mathew (1989) included *I. grijsi* and *I. speculatrix* in the addendum. Section Lophiris and subsection Evansia circumscribe the same species at different ranks. Dykes (1913) and Lawrence (1953) used the illegitimate name *I. minuta* Franch. & Sav. for *I. minutoaurea*.

Species	Dykes (1913)	Lawrence (1953)	Mathew (1989)	BIS (1997)
*I. cavaleriei* H.Lév.	Synonym of *I. grijsi*	Not included	Not included	Not included
*I. gracilipes* Pamp.	Not yet described	Not included	Not included	Synonym of *I. henryi*
*I. grijsi* Maxim.	Chinese group	Series *Chinenses*	Synonym of *I. speculatrix*	Synonym of *I. speculatrix*
*I. henryi* Baker	Chinese group	Series *Chinenses*	Series *Chinenses*	Series *Chinenses*
*I. koreana* Nakai	Not yet described	Series *Chinenses*	Series *Chinenses*	Series *Chinenses*
*I. minutoaurea* Makino	Chinese group	Series *Chinenses*	Series *Chinenses*	Series *Chinenses*
*I. odaesanensis* Y.N.Lee	Not yet described	Not yet described	Series *Chinenses*	Series *Chinenses*
*I. proantha* Diels	Not yet described	Not included	Sect. Lophiris Tausch	Series *Chinenses*
*I. rossii* Baker	Chinese group	Series *Chinenses*	Series *Chinenses*	Series *Chinenses*
*I. speculatrix* Hance	Section Evansia (Alef.) Baker	Subsect. Evansia Benth.	Series *Chinenses*	Series *Chinenses*

**Figure 1.**
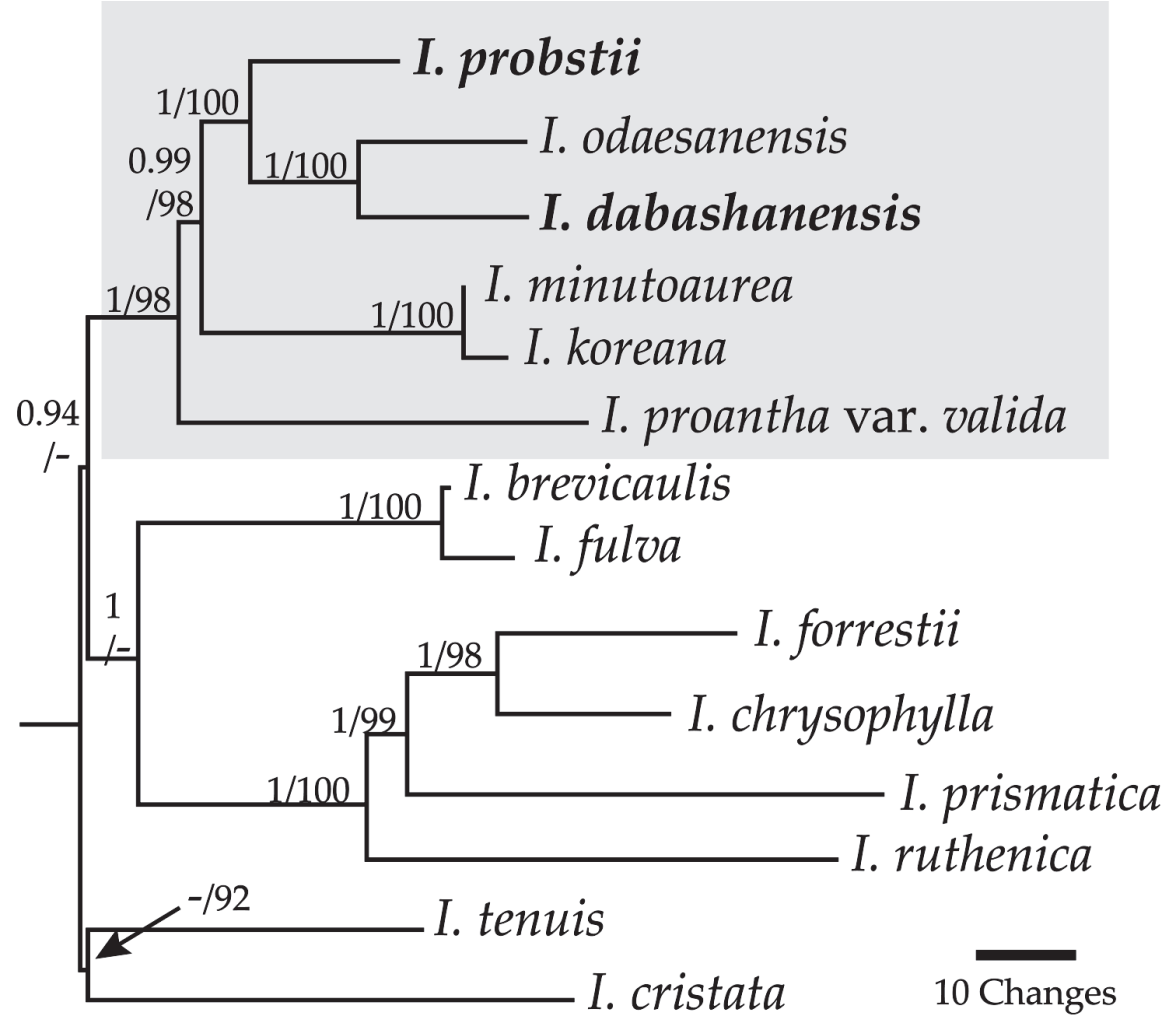
Phylogenetic tree showing resolution of *I. dabashanensis* and *I. probstii* (bold) in series *Chinenses* (grey box). Posterior probability/bootstrap values shown above branches.

Based on molecular studies (Tillie et al. 2001; Guo and Wilson 2013) and its morphology, including larger flowers and rhizomes, *I. speculatrix* is not considered a member of series *Chinenses* in this study. However, two taxa that are synonymized with *I. speculatrix* have not been recently examined. The first is *I. grijsi* Maxim. that was described based on a plant from Fujian Province in southeastern China. Maximowicz (1880) described *I. grijsi* as similar to *I. ruthenica* Ker Gawler, another species not resolved in series *Chinenses* (Wilson 2011; fig. 1), and gave several characters that distinguish it from *I. ruthenica*, including the presence of reduced leaves at the base of the flowering stem, a thicker rhizome, and inflorescences with three versus one flower. Collections assigned to *I. grijsi* are from central or eastern China. The second is *I. cavaleriei* H. Lév. from Guizhou Province (using the historic name, Kouy-Tchéou Province), the same province where one of the new species occurs. Léveillé (1905) noted the presence of narrow, short “interior” leaves and short vertical rhizomes in his recognition of the new species. Dykes (1913) interpreted the specimen of *I. cavaleriei* as having one elongated stem and a second flowering stem that was not yet visible, thus reducing the number of subtending smaller leaves, yet considered the taxon a synonym of *I. grijsi*.

In 2003, Darrell Probst collected one of the new species, *I. probstii*, at two sites in its native habitat in Guizhou Province and considered its flowers different from other species known from China. He sent materials of these plants to the author for further study. During this study it became apparent that the type specimen for *I. henryi* that was collected by Augustine Henry in 1885 and its description by Baker (1892) do not match the description of *I. henryi* in the Flora of China (Zhao et al. 2000) and most of the specimens identified as *I. henryi* that were studied by the author in Chinese herbaria in 2010. In addition, specimens at UC that were collected in 2001 by Darrell Probst in the Daba Mountains of northeastern Sichuan Province as *I. henryi* fit the description in Zhao et al. (2000) and are morphologically distinct from the type and description of *I. henryi*.

This study was undertaken to access the status of these two new species. The first goal was to determine morphological similarities and differences among these two new species, *I. dabashanensis* and *I. probstii*, and other species in series *Chinenses*. A second goal was to determine if species synonymized in *I. speculatrix* (*I. cavaleriei* and *I. grijsi*) are morphologically similar to *I. speculatrix* and distinct from each of the two new species. A third goal was to study materials of *I. henryi* and its illegitimate synonym, *I. gracilipes* Pamp., to determine if these two taxa are morphologically similar. *Iris gracilipes* Pamp. is illegitimate because the name was previously used by Asa Gray for a different species of Iris that is in section Lophiris Tausch (Gray 1858). A fourth goal was to use DNA sequence data to determine if *I. dabashanensis* and *I. probstii* are resolved in series *Chinenses*. A final goal was to comprehensively examine collections from southern China to identify previously collected specimens that represent these two new species.

## Materials and methods

### Morphological observations

Living specimens and vouchers of the two new species were examined and compared with each other. They were also compared to other species using descriptions, herbarium specimens, and images of herbarium specimens, focusing on species that occur in southern China and *I. odaesanensis*, a species resolved with molecular data in the same subclade as the two new species (Fig. 1). Herbarium sheets (BM, E, KUN, GH, P, PE, UC) and type descriptions of *I. cavaleriei*, *I. gracilipes* Pamp., *I. grijsi*, *I. henryi, I. odaesanensis* (type not seen), *I. proantha*, and *I. speculatrix* were compared to the new species. *Iris* specimens at KUN, PE, and UC were studied and photographed in May 2010 and E in May 2014. In July 2018 and October 2019 images of specimens were accessed and studied on the Natural History Museum data portal (BM; http://data.nhm.ac.uk), Harvard University Herbaria (A, ECON, GH; https://kiki.huh.harvard.edu/databases/image_search.php), and Muséum National d’Histoire naturelle vascular plant collection (P; https://science.mnhn.fr/institution/mnhn/collection/p/item/search). Images of specimens on the Chinese Virtual Herbarium (CVH; http://www.cvh.ac.cn/en) were accessed in September and October 2018 and August 2019 to study collections not previously seen, including a specimen of *I. odaesanensis* that was collected at the type locality and deposited in KUN after the visit in 2010. Collections of *I. gracilipes* Pamp. were not unearthed in these searches. In September 2019 materials of *I. gracilipes* Pamp. were requested from the herbarium at the Natural History Museum in Florence, Italy (Herbarium Universitatis Florentinae; FI) and six images were received, including the type. For this study, herbarium sheets or images of herbarium sheets of *I. cavaleriei* (1), *I. gracilipes* Pamp. (6), *I. grijsi* (23), *I. henryi* (54), *I. odaesanensis* (1), *I. proantha* (47), and *I. speculatrix* (322) were studied.

### Phylogenetic analyses

Genomic DNA of the new species, *I. probstii*, was isolated from silica-dried leaf materials using protocols modified from the CTAB method of Doyle and Doyle (1987). Modifications of this procedure included RNase treatment and an ethanol precipitation with ammonium acetate following the initial isopropanol precipitation. Plastid sequence data of the protein-coding *matK* gene, about 1,100 bp of the flanking *trnK* introns, and the intergenic spacer region *trnL–F* were generated and added to previously gathered data (Wilson 2009, 2011; Guo and Wilson 2013). The new species, *I. dabashanensis*, was included in previous studies (Wilson 2011; Guo and Wilson 2013) but at the time was considered *I. henryi*. DNAs were amplified in 25 ml reactions using GoTaq Flexi DNA polymerase (Promega, Madison, Wisconsin, U.S.A.) following the manufacturer’s instructions, except that 1–2 ml of glycerol was added to the mix. Reaction conditions were: 97 °C for 1 min; 40 cycles of 97 °C for 10 s, 48 °C for 1 min, 72 °C for 20 s; 72 °C for 4 min. The *matK* gene and flanking *trnK* introns were amplified together in two reactions while *trnL–F* was amplified in a single reaction. Amplification and sequencing primers are given in Wilson et al. (2016). Amplicons were purified using the UltraClean PCR clean-up kit (Mo Bio, Carlsbad, California, U.S.A.). Purified PCR products were processed using a BigDye Terminating (Applied Biosystems, Foster City, California, U.S.A.) cycle sequencing reaction following the manufacturer’s instructions except that 5% DMSO was added to the reaction mix. Cycle sequencing products were purified using filter plates packed with Sephadex (Amersham Biosciences, Piscataway, New Jersey, U.S.A.) and run on an Applied Biosystems 3130 automated sequencer. Molecular data was gathered by the author at the Rancho Santa Ana Botanic Garden, Claremont, California, U.S.A.

Geneious 9.1.4 (Biomatters Ltd., Auckland, New Zealand) was used to assemble the generated sequence reads into loci, concatenate loci by species, and align the final dataset. Equivocal nucleotide sites were coded according to the IUPAC (IUB) codes. Maximum likelihood (ML; Felsenstein 1981) and ML bootstrap (Felsenstein 1985) analyses were performed using RAxML-HPC v. 8 (Stamatakis 2014). Five hundred replicates for bootstrap and two for ML were performed. Bayesian inference (BI) was performed on the dataset using MrBayes v. 3.2.6 (Ronquist et al. 2012). Five replicates were performed for BI with the number of substitution rates set to six, the number of nucleotide frequencies set to four, the gamma distribution was approximated using four rate categories, and a proportion of the nucleotide sites was allowed to be invariable. The BI analyses were set to run for two million generations, each with four chains, and were sampled every 1000 generations. The final average standard deviations of split frequencies was < 0.005 and the potential scale reduction factor (PSRF) was 1.0 for all node and branch parameters indicating convergence. Estimated sample size (ESS) for each parameter was > 440. All phylogenetic analyses were performed on XSEDE accessed through the CIPRES portal (https://www.phylo.org). The resulting ML tree was exported from the CIPRES portal and modified in Adobe Illustrator vers. 15.0.0 (Adobe Inc., San Jose, California, U.S.A.) to include posterior probabilities and identify the series *Chinenses* clade.

### Distribution and mapping

Collection sites of specimens representing the two new species were located on Google Earth Pro vers. 7.3.2.5776 (Google LLC, Menlo Park, California, U.S.A.) to determine the known range of each species. Maps were produced in QGIS vers. 2.18.13 (QGIS Development Team 1991) using the free vector and raster map data available through Natural Earth (https://www.naturalearthdata.com). Maps were imported into Adobe Illustrator vers. 15.0.0 where species ranges were drawn.

### Data resources

The data underpinning the analyses reported in this paper are deposited in the Dryad Digital Repository at: https://doi.org/10.6078/D1C695

## Results

### Morphological comparisons

The two new species are morphologically most similar to *I. henryi* but differ in several characters as described below in the new species’ diagnoses. The two new species and *I. henryi* share several characteristics that distinguish them from *I. cavaleriei*, *I. grijsi*, and *I. speculatrix*, including smaller flowers, slender rhizomes that are also short, and long slender pedicels that result in exerted flowers and ovaries. The two new species and *I. henryi* also lack reduced basal leaves that are present in *I. cavaleriei* and *I. grijsi*. *Iris proantha* differs morphologically from the new species and *I. henryi* in several characters, including slightly larger flowers, longer floral tubes, and pedicels that are shorter and wider. Pedicels in *I. proantha* are more similar to those in *I. cavaleriei*, *I. grijsi*, and *I. speculatrix* than to the new species or *I. henryi*. Collections from the Daba Mountains and identified as *I. henryi* were determined to represent the new species, *I. dabashanensis*. Recent descriptions of *I. henryi* either fit *I. dabashanensis* (Zhao et al. 2000) or are broad enough to include this new species as well as *I. henryi* (Mathew 1989; BIS 1997).

Phylogenetic studies resolved the two new species in series *Chinenses* in a clade comprised of the new species + *I. odaesanensis* (Fig. 1). In the description of *I. odaesanensis*, Lee (1974) considered the species allied to *I. koreana* but differing in having longer rhizomes and white rather than yellow flowers. Study of the online image of a herbarium sheet at KUN and the species description for *I. odaesanensis* illustrated differences and similarities with the two new species. The leaves of *I. odaesanensis* are approximately 1 cm in width, similar to leaves of *I. koreana* and wider than the 0.1–0.2 and 0.4–0.6 cm leaves present in the new species, *I. dabashanensis* and *I. probstii*, respectively. In addition, *I. odaesanensis* does not occur in south-central China but rather is documented from Korea and is reported from adjacent areas in Jilin Province, northeastern China. The flowers of *I. odaesanensis* are white with yellow on the center of the sepals; a distinct color and patterning that is not seen in other Asian *Iris*. Several characters, including short floral tubes, long pedicels, and exerted flowers and ovaries are shared among *I. odaesanensis*, the two new species, and *I. henryi*.

Study of specimens and type descriptions revealed morphological differences among *I. cavaleriei*, *I. grijsi*, and *I. speculatrix*. The former two taxa are considered synonyms of *I. speculatrix*. Each of these three taxa are distinct from the two new species because they lack the long pedicels, and short floral tubes present in *I. dabashanensis* and *I. probstii* and have larger flowers and rhizomes than species in series *Chinenses*. Notes from examination of the *I. cavaleriei* holotype deposited at the Royal Botanic Garden Edinburgh (E) describes a plant with basal leaves that are 0.3 cm in width with two veins, unequal lower bracts (9 and 12 cm), flower exerted above bracts, ovary hidden by bracts, sepals that are ca. 2.5 cm long x 0.4 wide, and reduced leaves around the base of the flower stem. Photographs taken at E show an ovary enclosed in bracts with the visible portion of the floral tube greater than 1 cm. A more precise description of the floral tube requires re-examination of the type because this measurement was not included in notes taken at E and Léveillé (1905) did not include measurements when he described the species. Images of herbarium sheets of *I. grijsi* from CVH, KUN, P, and PE, including the type (P), were studied as well as its description. The leaves of *I. grijsi* appear wider than those of *I. cavaleriei* and are given as 0.4–1 cm in the description while the bracts are long and narrow, similar to *I. cavaleriei*. Dykes (1913) gave the bract length of *I. grijsi* as 2–3 in (ca. 5–7 cm). Neither the flower nor ovary of *I. grijsi* are exerted above the bracts, although the flower is visible because it extends beyond the shorter of the two bracts due to its floral tube that is ca. 1.2 cm in length. Similar to *I. cavaleriei*, reduced leaves are present at the plant base. In contrast to these two taxa, *I. speculatrix* lacks reduced basal leaves, has a shorter floral tube (< 1 cm), and short unequal bracts (ca. 2.5 and 3.5 cm). It also has a flower that is exerted above the bracts and an oblong ovary within or partially exerted above the bracts but visible because the bracts are spreading.

Comparisons between descriptions and herbarium sheets of *I. gracilipes* Pamp. and *I. henryi* did not reveal differences between these two species supporting the inclusion of the illegitimately named *I. gracilipes* Pamp. within *I. henryi*. However, it is possible that there are differences between these two taxa that are not evident in the known collections of *I. gracilipes* Pamp. These collections were made by Reverend Père Cipriano Silvestri in the early 1900s in northwestern Hubei Province. The type location of *I. henryi* is in central Hubei Province from the area of Liantuo (Nanto) west of Ichang (Yichang). Future fieldwork in the region of Silvestri’s collections and the type location of *I. henryi* is planned to further investigate these taxa. *Iris henryi* was not located during fieldwork by a colleague in 2019 west of Yichang, leading to concern that the type location may have been lost with development of the Three Gorges Dam on the Yangtze River.

### Additional collections of the new species

No additional collections of the new species from Guizhou Province, *I. probstii*, were identified during study of herbarium specimens and images of specimens, although three specimens from Guizhou Province were examined. One specimen identified as *I. speculatrix* was collected in 2013 and deposited at the Guizhou Academy of Sciences (HGAS; 123147). Two specimens identified as *I. grijsi* were collected in 1898 and 1900 and deposited at the Muséum National d’Histoire (P; 01840535 and 0184536, respectively). Study of the image of the former specimen revealed that it fit the description and type of *I. speculatrix*. Images of the two specimens identified as *I. grijsi* were also studied and are most similar to other collections of *I. grijsi*. Specimen 01840535 is particularly informative, although a scale was not included when imaged. This specimen illustrates the short leaves at the base of the plant that are typical for this taxon and the longer bracts when compared to *I. speculatrix*.

The study of herbarium records revealed 34 additional specimens from the Daba Mountains that are the new species, *I. dabashanensis*, but were identified as other species. These represent sheets from nine separate collections from the Chongqing Municipality and one from the Shennongjia Forest Region in Hubei Province. Three botanists, Père Paul Guillaume Farges in 1892, Tain Lun Dai in 1958, and Du Wei in 2014 made these collections. Eight of the collections were identified as *I. henryi* while the 2014 collection was identified as *I. proantha*. It is not surprising that these collections were mostly identified as *I. henryi* because *I. dabashanensis* is similar to this species and in particular has long slender pedicels and flowering stems that are shorter than leaves at flowering, two characters considered distinguishing for *I. henryi*. A few other collections from near the Daba Mountains may also represent *I. dabashanensis* but a determination could not be made because flowers were lacking or important characters were not clearly visible.

### Phylogenetics

The aligned, combined dataset was 3,551 bp with 398 (11%) variable and 153 (4%) potentially parsimony informative characters. The *matK*/*trnK* marker was more informative than *trnL–F* because it contributed 2,696 bp with 322 variable and 114 potentially parsimony informative characters. The resulting models from RAxML and MrBayes analyses differed only slightly in the three substitution rate and gamma distribution values (the numbers given are based on RAxML). Both assumed unequal base frequencies, three substitution rates (1.0000, 2.8325, 0.1513), a gamma distribution of 0.5726 and 0.3177 invariable sites. The single ML tree from RAxML and the consensus tree from MrBayes each had a log likelihood = -7,779 and shared the same topology (Fig. 1). Posterior probability and bootstrap values are given in Figure 1.

The two new species were resolved with high support in a clade with *I. odaesanensis*. As described above, these three species share several morphological characters, including long slender pedicels and short floral tubes. They were resolved with high support within the series *Chinenses* clade and share several characters with other species in the series, including small, horizontally spreading flowers where petals are not upright, an overall small and delicate stature, short rhizomes, and an Asian distribution. All taxa in series *Chinenses*, except *I. henryi*, the nominative form of *I. proantha*, and *I. rossii*, were included in this study. Further studies to resolve relationships within the series should include these three taxa and also *I. grijsi*, *I. cavaleriei*, and *I. speculatrix*. The former two taxa are considered synonyms of *I. speculatrix* but in this study were shown as morphologically distinct. The type locality for *I. henryi* is not precise and the species has not been relocated in the area of its initial discovery.

### Other findings

The study also resulted in the discovery of two type collections and one correction to a species description citation. The type of *I. speculatrix* (C. Ford 18465) was discovered in BM collections stored as a type of *I. henryi* and the type of *I. grijsi* (de Grijs 8583) in P collections but not designated as a type. The description of *I. cavaleriei* is typically cited as Liliac. & C. Chine 18 1905, but previous searches for this journal were not successful. During this study the author searched journals where Augustin Abel Hector Léveillé published species descriptions, focusing on journals where he published infrequently. The correct citation is given in the references (Léveillé 1905). In this publication Léveillé lists Iridaceae known from China and provides a description of *I. cavaleriei* sp. nov. citing the collection of Julien Cavalerie from Kouy-Tcheou (Guizhou) Province on May 15, 1900.

### Taxonomy

#### Iris dabashanensis

Taxon classificationPlantaeMantodeaTarachodidae

C.A.Wilson sp. nov.

70F7C51E-1F0F-5B01-B935-E283C1C62D96

urn:lsid:ipni.org:names:77211598-1

Figures 2A–C , 3

##### Diagnosis.

Morphologically similar to *I. henryi* the new species differs in having narrower leaves (0.9–2 mm versus 4–4.5 mm), shorter bracts (2–4 cm versus 4–7 cm), and shorter style branches (1 cm versus 1.8 cm).

##### Type.

China. Sichuan Province: About 5 km N of Wanyuan, Wanyuan County, 1,140 m, 32°11.720'N, 108°05.310'E, 3, May 2001 (fl), voucher from cultivated material, *D. Probst CPC3.5.01.3* (***holotype***: UC!; ***isotypes***: PE!, E!).

**Figure 2.**
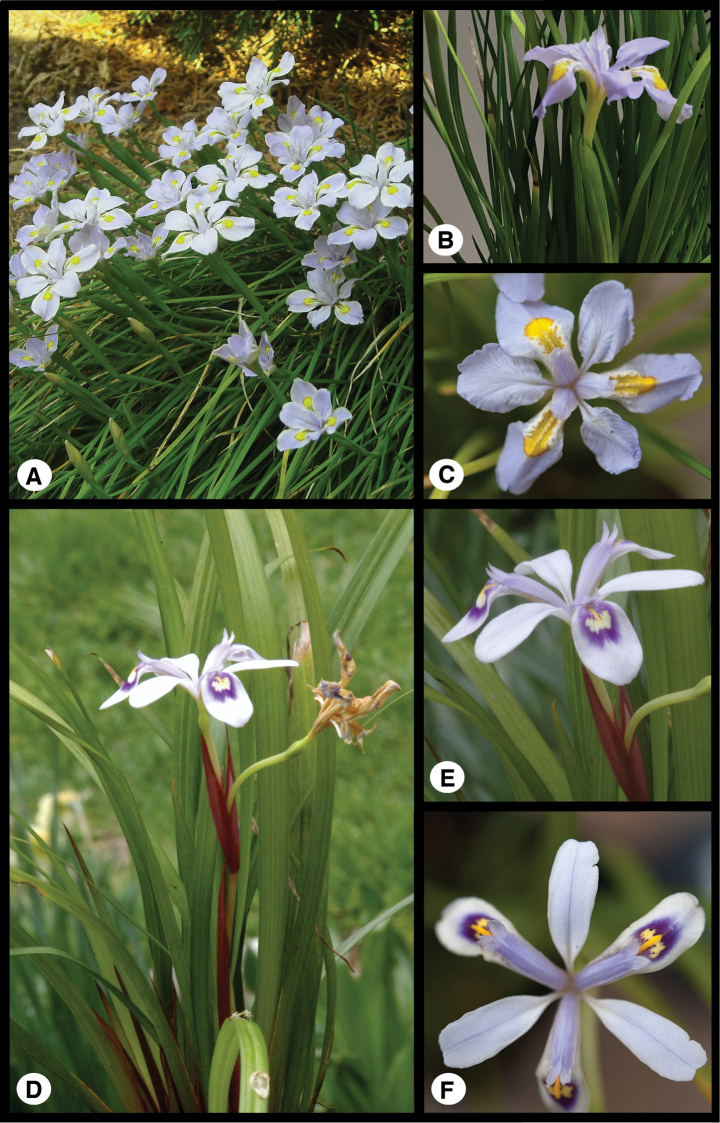
Images **A–C***I. dabashanensis***D–F***I probstii*. **A** Habit **B** inflorescence **C** flower **D** habit **E** inflorescence **F** flower (Photos **B–F** C. author; **A** Mark McDonough).

##### Description.

Small evergreen herbaceous plant with aerial flowering stems less than basal leaves. Bracts, cauline leaves, base of basal leaves ± tinged with red. Rhizomes short and branched with fibrous leaf remains. Stolons present. Roots slender, branched, with tubers to 6 mm. Basal leaves distichous, linear with acute apex, bright green, slightly glossy, 28–44 cm long, 0.9–2 (3) mm wide, 1–2 veins; crowded in clumps. Flowering stem ca. 8–18 cm with 1 or 2 cauline leaves, with upper cauline leaf extending well above mid stem. Inflorescence with two opposite bracts (spathes) subtending 2 (3) flowers, terminal flower opening first with a single bract subtending each subsequent flower; lower bracts ± equal, 2.0–4 cm long, 0.4–0.5 cm wide; pedicels long (2.5–5 cm) and slender. Flowers light violet, open, ca. 3 cm diameter, radial with petals slightly angled upward, sepals light violet, horizontal and recurved distally; floral tube 0.2–0.5 cm; sepals ovate, shallow apical notch, narrowed at base, 2–3 cm long, 0.6–1.1 cm wide, obvious median ridge cream-colored with violet spots, a lateral ridge on either side of the median, yellow patch between lateral ridges and extending beyond ridges; petals light violet, rotund, apical notch, 1.6–2.1 cm long, ca. 1.0 cm wide, clawed in lower 1/4; stamens cream, ca. 0.8 cm long, anthers = filaments; style branches petaloid, light violet, ca. 1 cm long, 0.3 cm wide, bi-lobed distally, lobes ca. 0.4 cm long, stigma broad, slightly rounded. Capsule rounded, ca. 1 cm long. Seed light to medium brown, ca. 0.4 cm, with conspicuous white appendage. Flowering: April to May.

**Figure 3.**
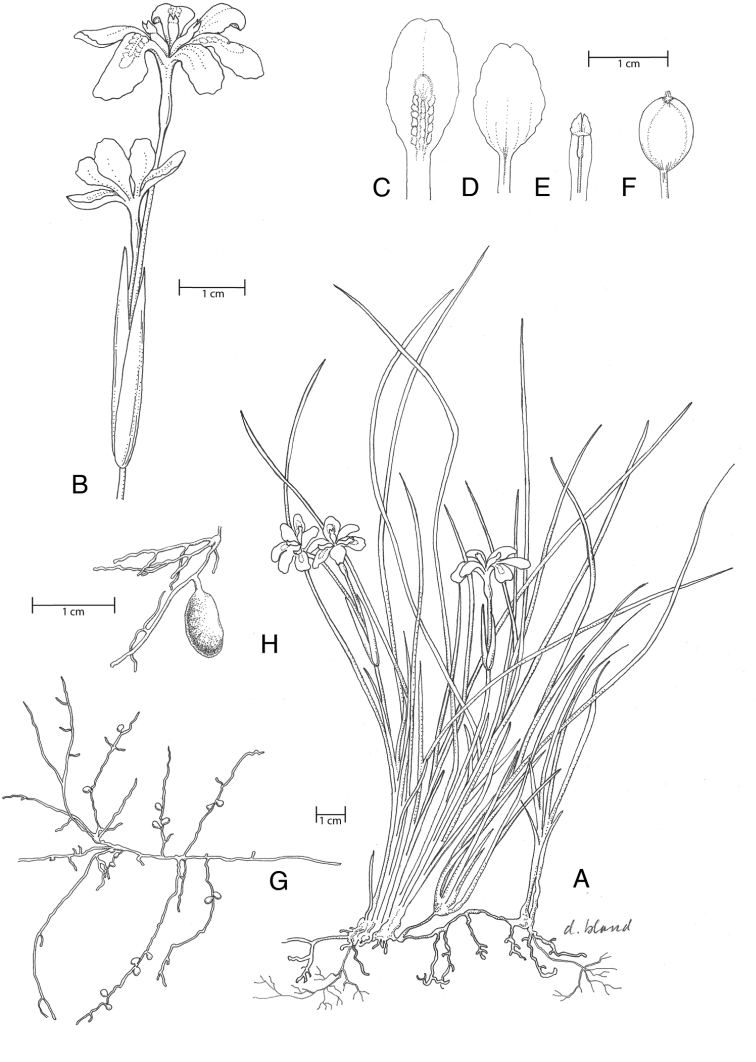
Illustration of *I. dabashanensis*. **A** Habit **B** inflorescence **C** sepal **D** petal **E** style branch, anther **F** fruit **G** stolon, roots, tubers **H** root tuber (Source: **A–E***D. Probst CPC3.5.01.3* (UC); **F** photograph by Marty Schafer and Jan Sacks; **G, H** photograph by D. Probst, photographs available from author).

##### Distribution and ecology.

**Figure 4.**
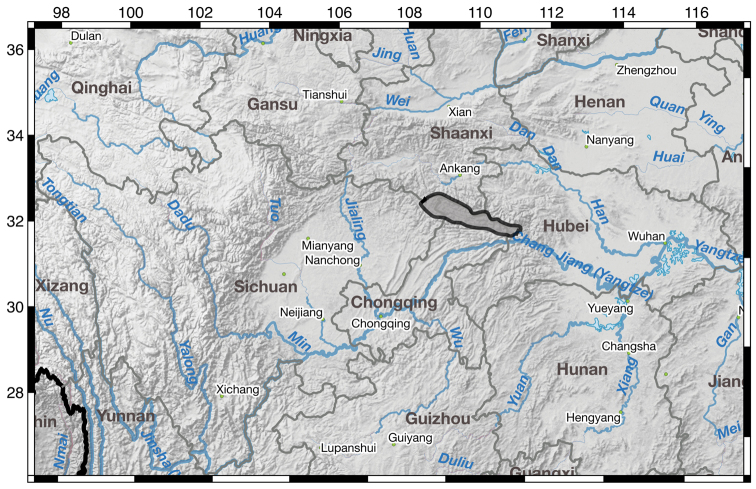
Map of known distribution for *I. dabashanensis* in gray outlined in black.

*Iris dabashanensis* is known from the Daba Mountains in Sichuan and Hubei Provinces and Chongqing Municipality (Fig. 4) on open rocky slopes with calcareous soils associated with shrubs or mixed conifer and hardwood forests.

##### Etymology.

The new species is named for the Daba Mountains in China where it occurs.

##### Preliminary conservation status.

Following the criteria and categories of IUCN (2012), *Iris dabashanensis* is given a preliminary status of Least Concern (LC). One of the known populations is in the Shennongjia Forest Region of Hubei Province. Although the precise location of the *I. dabashanensis* population is unknown, the Shennongjia National Nature Reserve occurs within the region and has protected status.

##### Other specimens examined.

China. 4 ⚥ Chongqing Municipality, Chengkou County; 29 April 1958; *T.L. Dai* leg.; *100265*; HNWP 34156, PE 01012365, SZ 00043052, SZ 00043057; · 5 ⚥ Chongqing Municipality: Chengkou County; 1 May 1958; *T.L. Dai* leg.; *100335*; CDBI 0169543, IBK 00251078, SZ 00043056, PE 00034001, PE 01012364; · 4 ⚥ Chongqing Municipality: Liangzhong Hewu ji, Chengkou County; 20 April 1958; *T.L. Dai* leg.; *100150*; PE 00034002, SZ 00043055, HNWP 34209, IBK 00251010; · 1 ⚥ Chongqing Municipality: Liang Yizh, Chengkou County; 2 April 1958; *T.L. Dai* leg.; *100154*; CDBI 0169546; · 6 ⚥ Chongqing Municipality: Yizi Liang Hengyan pengwuji, Chengkou County; 24 May 1958; *T.L. Dai* leg.; *100583*; IBK 00081641, CDBI 0169544, CDBI 0169545, HNWP 34583, SZ 00043061, PE 01012366; · 1 ⚥ Chongqing Municipality: Chengkou County; sin. date; *Farges* leg.; *101*; P 02163317; · 4 ⚥ Chongqing Municipality: Chengkou County; 1892; *Farges* leg.; *1024*; P 02163312, P 02163313, P 02163314, P 02163316; · 11 ⚥; Hubei Province: Shennongjia Forest Region; 27 April 2014; *Du Wei* leg.; *1407*; WH 1933.

##### Notes.

The date on the specimen *T.L. Dai 100154* was 1918 but the collections made at the same time and place had a date of 1958 which is consistent with Dai’s numbering of specimens and other collections.

#### Iris probstii

Taxon classificationPlantaeMantodeaTarachodidae

C.A.Wilson sp. nov.

839FE4C0-A63B-5D47-8308-F578340B6DEE

urn:lsid:ipni.org:names:77211599-1

Figures 2D–F , 5

##### Diagnosis.

Morphologically similar to *I. henryi*, the new species differs in having wider sepals (ca. 7 mm versus 4 mm) and style branch lobes that are shorter (ca. 2–3 mm versus 6 mm).

##### Type.

China. Guizhou Province: about 24 km south of Yanhe on road to Xiushan, 970 m, 28°25.643'N, 108°42.044'E, 24, July 2003 (fl), voucher from cultivated material, *D. Probst CPC24.7.03.1* (**holotype**: UC!; **isotypes**: PE!, E!).

##### Description.

Small evergreen herbaceous plant with aerial flowering stems shorter than basal leaves. Bracts, cauline leaves, base of basal leaves ± tinged with red. Rhizomes short (< 4 mm), branched, hidden by leaf remains. Stolons present. Roots highly branched distally with small tubers. Basal leaves distichous, linear with acute apex, bright green, slightly glossy on upper surface, dull green on lower surface, leaf edges obscurely membraneous, 12–40 cm long, 0.4–0.6 cm wide, veins slightly thickened; crowded in clumps. Flowering stem ca. 15 cm with 2 (3) cauline leaves on lower half. Inflorescence with two opposite bracts (spathes) subtending 2 (3) flowers, terminal flower opening first with a single bract subtending each subsequent flower; lower bracts ± unequal, 3.5–6 (7) cm long, 0.4–0.5 cm wide; pedicels long (3–4.5 cm) and slender. Flowers cream with light violet, open, ca. 3 cm diameter, radial with petals slightly angled upward, sepals horizontal and recurved distally; floral tube 0.3–0.4 cm; sepals cream adaxially, light yellow abaxially, narrowly obcordate, ± shallow apical notch, narrowed at base, 2.2–2.9 cm long, 0.6–0.9 wide, obvious yellow-orange median ridge, purple spot beginning at ca. midpoint and extending distally beyond ridge with two bright yellow patches flanking ridge distally; petals cream with light violet tint that is more prominent along median, narrowly spathulate, 1.8–2.2 cm long, 0.6–0.7 cm wide, clawed in lower 1/4; stamens cream, ca. 1 cm long, anthers = filaments; style branches medium violet, 1.2–1.5 cm long, 0.3–0.4 cm wide, bi-lobed distally, light violet lobes ca. 0.2–0.3 cm long with several teeth, stigma broad, slightly rounded. Capsule rounded with short apical beak. Seed light to medium brown, ca. 0.4 cm, with conspicuous white appendage. Flowering: April to May.

**Figure 5.**
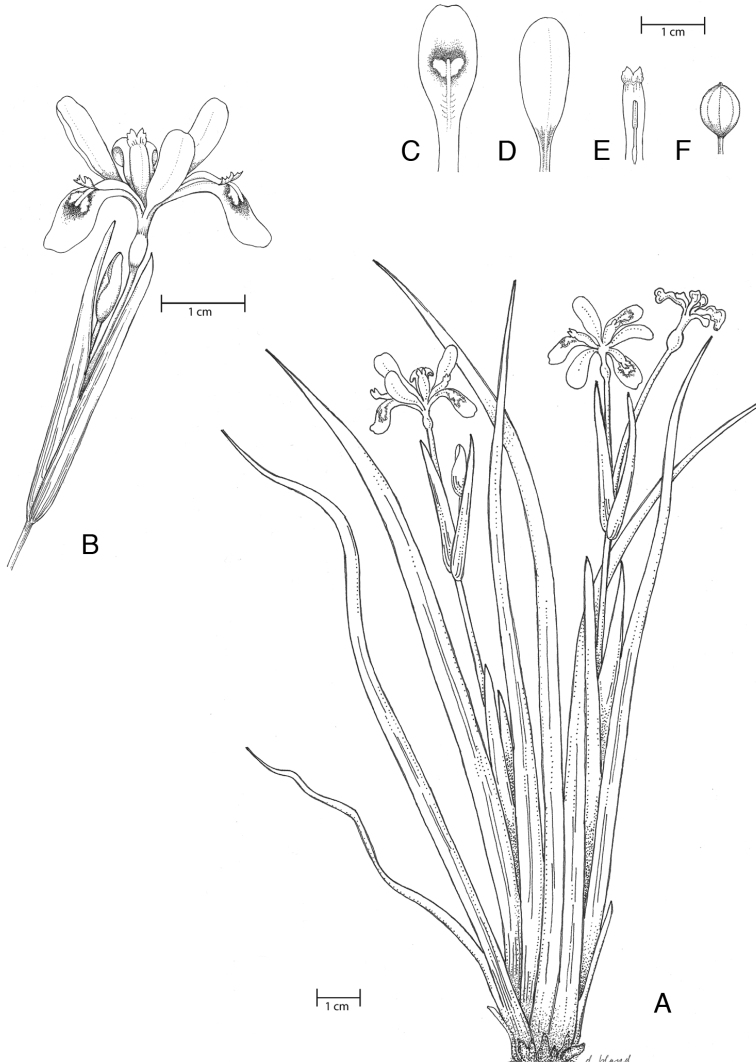
Illustration of *I. probstii*. **A** Habit **B** inflorescence **C** sepal **D** petal **E** style branch, anther **F** fruit (Sources: **A–E***D. Probst CPC24.7.03.1* (UC); **F** photograph by M. Schafer and J. Sacks, photographs available from author).

##### Distribution and ecology.

**Figure 6.**
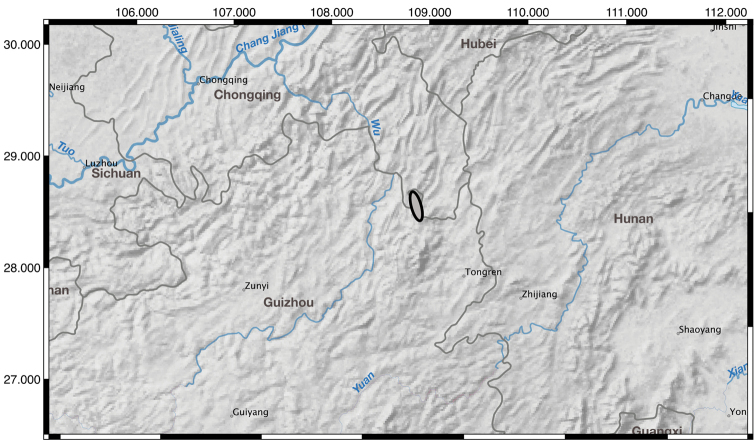
Map of known distribution for *I. probstii* in gray outlined in black.

*Iris probstii* is currently known from two locations south of Yanhe in Guizhou Province China (Fig. 6) at about 800 to 1,000 m and a third unverified location about 165 km south of Yanhe. This third site, southeast of Tongren, Guizhou, China near Minhezhen (Minhe town), is based on photographs taken by C. Yang in 2016 and uploaded to the Plant Photograph Bank of China (http://ppbc.iplant.cn/tu/6166544). A study of herbarium records from CVH, KUN, and PE did not reveal additional collections but it is likely that this new species occurs in other areas within this region. The species occupies open slopes along edges of rice paddies or under low pine woods in grassy sites associated with rocky, karst soils.

##### Etymology.

The new species is named in honor of the U.S. horticulturist, Darrell Probst, in recognition of his work to expand our knowledge of species from series *Chinenses* in their native habitats.

##### Preliminary conservation status.

Following the criteria and categories of IUCN (2012), *Iris probstii* is given a preliminary status of Vulnerable (VU D2) due to its restricted area of known occupancy (< 20 km^2^) and number of locations (< 5). The area of occurrence does not have protected status. If the third location is verified and vouchered the conservation status might change.

##### Other specimens examined.

China. Guizhou Province: about 55 km south of Yanhe on road to Xiushan, 825 m, 28°19.651'N, 108°41.390'E, 9, January 2002 (fl), *D. Probst CPC9.1.02.2* (photo of living plant, UC).

##### Notes.

D. Probst collected a small rhizome segment at the second site that he grew but did not voucher. An image of the plant was obtained for documentation and deposited in UC. A specimen voucher will be made and also deposited at UC when the plant reflowers.

## Discussion

Morphologically these two new species are most similar to *I. henryi* because they share several characters, including long (> 2.5 cm) slender pedicels, short floral tubes, and narrow leaves (< 0.6 cm). *Iris odaesanensis* shares the characters of short floral tubes and long pedicels with the two new species and *I. henryi* but it has leaves that are about 1 cm in width. Molecular data indicates that the two new species are closely related to *I. odaesanensis*. Unfortunately, because materials of *I. henryi* were not available for molecular studies it is unknown if all series *Chinenses* species with short floral tubes and long pedicels are resolved in a clade. These four species can easily be distinguished from each other using several characters. *Iris odaesanensis* has flowering stems that are almost equal or slightly longer than leaves at flowering while *I. dabashanensis*, *I. henryi*, and *I. probstii* have flowering stems that are substantially shorter than their leaves at flowering. *Iris dabashanensis* has very narrow (ca. 1–2 mm) leaves while the width of *I. henryi* and *I. probstii* leaves are similar at 4–6 mm. *Iris dabashanensis* also has a median and two lateral ridges while *I. henryi*, *I. odaesanensis*, and *I. probstii* have only a median ridge. *Iris probstii* has style branches where lobes are 0.2–0.3 cm long which is ca. 1/4 of their total length while lobes in *I. dabashanensis* and *I. henryi* are > 0.4 cm and ca. 1/2 of their total length.

Both *I. dabashanensis* and *I. probstii* occur in areas that are rocky and considered limestone rich. The border area between Chongqing Municipality and Guizhou Province south of Yanhe, where *I. probstii* occurs, is considered a karst region with a deep layer of limestone and dolomite and characteristic landforms of caves, sinkholes, outcrops, natural bridges, and gorges. Guizhou Province is one of four provinces that make up the South China Karst, UNESCO World Heritage Site. The Daba Mountains are also rich in limestone and on the northwestern slopes of the Daba Mountains in Shaanxi Province there is a large region of karst. However, the southern slopes of the Daba Mountains above the Sichuan Plateau where *I. dabashanensis* occurs is not considered a karst region although the soils are calcareous.

An examination of available materials suggests that *I. cavaleriei* and *I. grijsi* are similar to each other but morphologically distinct from *I. speculatrix*. They are also morphologically distinct from the two new species and other series *Chinenses* species. Previous research utilizing molecular data (Tillie et al. 2001; Guo and Wilson 2013) concluded that *I. speculatrix* is not within series *Chinenses*, a finding consistent with the larger flowers and rhizomes of *I. speculatrix* compared to species in series *Chinenses*. The larger flowers and rhizomes of *I. speculatrix* are shared with *I. cavaleriei* and *I. grijsi*. The status of *I. cavaleriei*, which was described after *I. grijsi*, is not certain although results of this study indicate leaves of *I. cavaleriei* are narrower than those of *I. grijsi* and the flower is only partially exerted above the bracts while it is entirely exerted in *I. grijsi*. These observations are based on only one collection, the holotype, of *I. cavaleriei*. Examining additional plants from the type locality for *I. cavaleriei* and/or the identification of additional existing collections is required to determine if the differences observed in the one known collection are consistent within the taxon and if the taxon is distinct from *I. grijsi*.

It is surprising that during this study of over 450 specimens representing six *Iris* species with collections in southern China, only three were collected in Guizhou Province. Guizhou Province is a mountainous rural region that is recognized as having high plant diversity (Zhang and Ma 2008; Liu et al. 2018) and endemism (Liu et al. 2018). The karst landscape and ecology is considered an important contributor to the high biodiversity of the Province. *Iris* may be undercollected within the region indicating that further fieldwork is necessary to more fully understand *Iris* diversity in the Province and the entire range of *I. probstii*.

### Key to *Iris* series *Chinenses*

**Table:** 

1	Leaves > 1 cm wide	**2**
–	Leaves < 1 cm wide	**3**
2	Floral tube ca. 1 cm long, flowers yellow	***I. koreana***
–	Floral tube ca. 0.5 cm long, flowers white	***I. odaesanensis***
3	Floral tube > 2.0 cm long, pedicels < 2 cm long	**4**
–	Floral tube < 1.0 cm long, pedicels > 2 cm long	**6**
4	Floral tube ca. 6 cm long	***I. rossii***
–	Floral tube 2–3.5 cm long	**5**
5	Flowers pale violet, floral tube 2.5–3.5 cm long	***I. proantha***
5a	Flowering stem to bract < 5 cm tall, leaves < 0.4 cm wide	**var. proantha**
–	Flowering stem to bract > 7 cm tall, leaves > 0.5 cm wide	**var. valida**
–	Flowers yellow, floral tube ca. 2 cm	***I. minutoaurea***
6	Leaves < 3 mm wide, lateral crests flanking median	***I. dabashanensis***
–	Leaves > 3 mm wide, median crest only	**7**
7	Style branch lobes 2–3 mm long, sepals ca. 7 mm wide	***I. probstii***
–	Style branch lobes ca. 6 mm long, sepals ca. 4 mm wide	***I. henryi***

## Supplementary Material

XML Treatment for Iris dabashanensis

XML Treatment for Iris probstii

## Supplementary materials

Supplementary material 1 Supplementary file for Dryad Data type Nucleotide dataset, phylogenetic tree Explanation note This is a draft file for submission to Dryad as supporting materials. https://binary.pensoft.net/file/452701This dataset is made available under the Open Database License (http://opendatacommons.org/licenses/odbl/1.0/). The Open Database License (ODbL) is a license agreement intended to allow users to freely share, modify, and use this Dataset while maintaining this same freedom for others, provided that the original source and author(s) are credited.Carol A. Wilson
